# Hepatitis B Virus HBx Protein Interactions with the Ubiquitin Proteasome System

**DOI:** 10.3390/v6114683

**Published:** 2014-11-24

**Authors:** Marissa M. Minor, Betty L. Slagle

**Affiliations:** 1 Graduate Program in Molecular Virology and Microbiology, Baylor College of Medicine, Houston, TX 77030, USA; E-Mail: marissa.minor@bcm.edu; 2 Department of Molecular Virology and Microbiology, Baylor College of Medicine, Houston, TX 77030, USA

**Keywords:** hepatitis B virus, HBx, ubiquitin, DDB1, CSN, proteasome

## Abstract

The hepatitis B virus (HBV) causes acute and chronic hepatitis, and the latter is a major risk factor for the development of hepatocellular carcinoma (HCC). HBV encodes a 17-kDa regulatory protein, HBx, which is required for virus replication. Although the precise contribution(s) of HBx to virus replication is unknown, many viruses target cellular pathways to create an environment favorable for virus replication. The ubiquitin proteasome system (UPS) is a major conserved cellular pathway that controls several critical processes in the cell by regulating the levels of proteins involved in cell cycle, DNA repair, innate immunity, and other processes. We summarize here the interactions of HBx with components of the UPS, including the CUL4 adaptor DDB1, the cullin regulatory complex CSN, and the 26S proteasome. Understanding how these protein interactions benefit virus replication remains a challenge due to limited models in which to study HBV replication. However, studies from other viral systems that similarly target the UPS provide insight into possible strategies used by HBV.

## 1. Introduction

Ubiquitin-mediated proteolysis is the major mechanism by which cells degrade proteins no longer needed. The highly conserved Ubiquitin Proteasome System (UPS) degrades proteins through the covalent attachment of the 76 amino acid (aa) ubiquitin (Ub) to target substrates. This results in either a modified function (for proteins that are monoubiquitinated) or protein degradation through the 26S proteasome (for proteins that are polyubiquitinated) [1,2,3]. This mechanism of protein degradation is important in a variety of cellular functions, including cell cycle regulation, apoptosis, transcriptional regulation, chromatin remodeling, DNA repair, and protein quality control (reviewed in [4]). There is a growing appreciation for the importance of the UPS to virus replication. Several viruses target the UPS as part of their strategy to benefit virus replication (reviewed in [5,6,7,8,9]).

The human hepatitis B virus (HBV) is a major cause of acute and chronic viral hepatitis. Worldwide, over 450 million people have chronic HBV infection and are at risk for the development of hepatocellular carcinoma (HCC) (reviewed in [10,11]). HBV is a small DNA virus with a total genome size of less than 3.2-kb. Due to its limited genetic information, it is likely that HBV relies heavily on the cellular pathways to provide functions needed for virus replication. The purpose of this review is to summarize the reported interactions of the HBV regulatory HBx protein with the UPS, and to discuss the impact of these interactions on virus replication and pathogenesis.

## 2. The UPS

**Figure 1:**
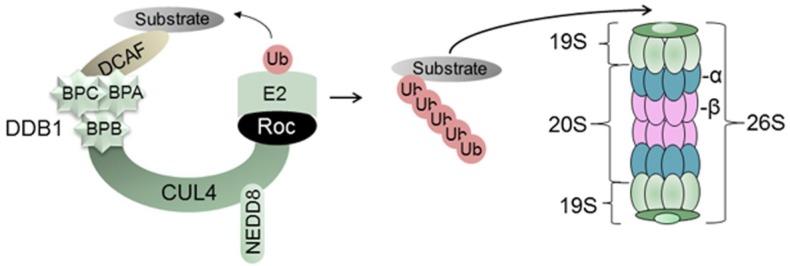
Schematic of the UPS. In the example provided, the E3 ligase consists of the CUL4 scaffold, the DDB1 adaptor protein (folded into three β-propellar, or BP, domains), and the RING protein (Roc) that binds to the E2 enzyme. This complex recruits substrate proteins for ubiquitination and degradation by the 26S proteasome, as described in the text.

The UPS is a conserved pathway in the cell that targets proteins for ubiquitination and proteolysis. The covalent modification of target proteins by Ub occurs via an enzymatic cascade that includes a Ub-activating enzyme (E1), a Ub-conjugating enzyme (E2), and a Ub-ligase enzyme (E3) that both recruits protein substrates and mediates the attachment of Ub (reviewed in [12]). The human genome encodes an E1 protein, ~40 E2 proteins, and several hundred E3 ligases (reviewed in [13]), thus providing a comprehensive ability to regulate diverse cellular pathways (reviewed in [4]). The most abundant E3 ligases are the Cullin-RING-ligases (CRLs), accounting for 95% of all E3 ligases. Mammals have seven highly conserved Cullin proteins (CUL1, CUL2, CUL3, CUL4A, CUL4B, CUL5, and CUL7) [14]. The CRL4 consists of the CUL4 scaffold, the Damaged DNA Binding Protein 1 (DDB1) adaptor at its amino end, and a RING (Really Interesting New Gene) protein at its carboxyl end (Figure 1). CUL4, the topic of this review, exists as two highly-related proteins, CUL4A and CUL4B that are 83% identical and functionally redundant [15]. In this review, the two proteins are collectively referred to as CUL4, and the E3 ligase complex in which they assemble is called CRL4.

### 2.1. Damaged DNA Binding Protein 1 (DDB1) and DDB1-Cullin-Associated Factors (DCAFs)

Damaged DNA binding protein 1 (DDB1) is a multifunctional protein originally known for its involvement in the recognition of UV-damaged DNA and the recruitment of nucleotide excision repair (NER) machinery to the site of damage (reviewed in [15,16,17]). Important for this review, DDB1 also functions as adaptor protein for the CRL4 complex (Figure 1). DDB1 recruits a subset of WD40 repeat proteins, termed DDB1 Cullin Accessory Factor (DCAFs) that bind to a specific region on DDB1 and recruit substrates to CRL4 for ubiquitination and proteasomal degradation [18,19,20] (Figure 1). The human genome encodes approximately 79 DCAFs that bind to the same region of DDB1 through a shared motif that is based on the biophysical properties of the amino acids rather than the primary amino acid sequence [18,19,21]. CRLs are also a frequent target of viruses that seek ways to manipulate the cell to benefit their own replication (reviewed in [6]). While this paper focuses on HBV interactions with CRL4, HBx is also reported to interact with the CUL1-SKP2 E3 ligase complex [22].

### 2.2. Regulation of CRLs

The CRLs provide important post-translational regulation of proteins, but are also themselves subject to negative regulation when their activities are not needed. All cullins are covalently modified by NEDD8 (neural precursor cell-expressed developmentally downregulated protein 8) (Figure 1), a small protein that is 76% identical to ubiquitin [23]. NEDD8 is required for CRL activity, and removal of NEDD8 is accomplished by the CSN5 subunit of the COP9 signalosome (CSN) (reviewed in [4,24]). Indeed, the CSN can be immunoprecipitated with CRL4 [25]. CRL4 may additionally be regulated through different isoforms generated by alternative splicing [26]. Some of the spliced CUL4s lack the first 100 aa [26] and are predicted to lose the ability to bind DDB1 [27]. Finally, the cullin-associated and neddylation-disassociated 1 (CAND1) protein and DDB1 compete for binding to CUL4 in a mutually exclusive manner [27]. CAND1 binds only with the unneddylated (inactive) form of CUL4 [27].

### 2.3. The 26S Proteasome

The final component of the UPS is the 26S proteasome, a large, multi-subunit complex found in both the nucleus and the cytoplasm (reviewed in [28,29]). The proteasome consists of a proteolytic core particle known as the 20S proteasome and two 19S regulatory particles (Figure 1). Tissue-specific versions of the 20S proteasome exist [30]. The proteasome recognizes ubiquitinated target proteins, and degrades them through its cylindrically-shaped 20S proteasome which contains 28 protein subunits organized into four stacked rings in which the outer rings are formed of seven α-subunits and the two inner rings are formed of seven β-subunits. Three of the seven β-subunits (β1, β2, β5) are involved with the enzymatic activities of the proteasome, which have been identified as trypsin-like (targeting arginine or lysine), chymotrypsin-like (targeting the carboxyl terminus of tyrosine, tryptophan and phenylalanine), and peptidylglutamyl-peptide hydrolyzing-like (cleavage after acidic amino acids) (reviewed in [28,29]). The 19S regulatory complex is made up of 20 subunits that attach to both sides of the 20S proteasome and is involved in ATP-dependent substrate degradation by binding to and removing the polyubiquitin chains by deubiquitin (DUB) enzymes, unfolding the substrate, and opening the channel of the core particle to allow substrate entry.

### 2.4. The UPS and Cancer

The cell cycle is carefully regulated by actions of the UPS, which controls apoptotic proteins, cyclins, and cell cycle inhibitors (reviewed in [2]). It is not surprising that deregulation of the UPS may play a role in the development of cancer. The *Cul4A* gene is located on human chromosome 13q34 and this region is amplified in several cancers (reviewed in [15]), including liver cancer [31]. Elevated expression of CUL4A is correlated with significantly shorter overall survival and accelerated neoplastic transformation in ovarian tumors and node-negative breast cancers [32,33] suggesting that elevated expression of CUL4A may promote carcinogenesis. Consistent with this, knockdown of CUL4A leads to inhibition of cancer cell growth and apoptosis, indicating that CUL4A may be a promising target for anti-cancer therapies (reviewed in [15]). In addition, the proteasome has served as a target for anti-cancer therapy. Actively dividing cells are more sensitive to proteasome inhibition than non-cancerous cells (reviewed in [28]). The U.S. Food and Drug Administration approved the proteasome inhibitor Velcade^®^ (bortezomib) for the treatment of multiple myeloma and relapsed mantle cell lymphoma [34,35]. However, these inhibitors are likely to have toxic effects on normal cell function. Targeting a specific protein in the UPS pathway, and perhaps a specific CRL, may be a more efficient way to maximize treatment while minimizing toxicity.

## 3. The HBV Life Cycle and HBx

### 3.1. HBV Replication

**Figure 2:**
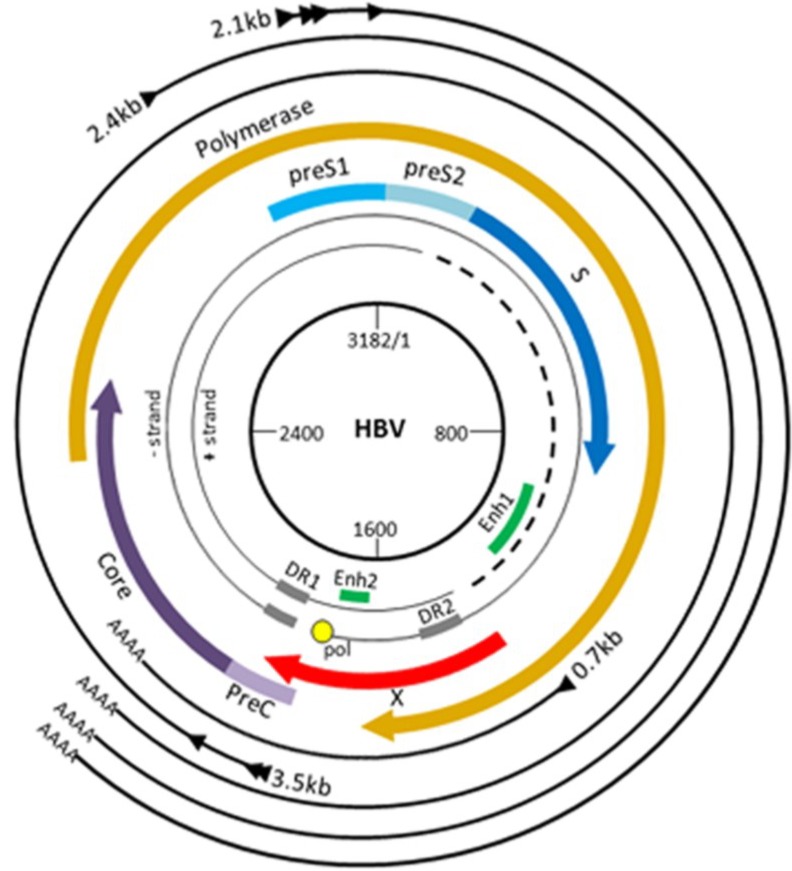
HBV genome organization. HBV contains a small, partially double-stranded (~dsDNA) genome (see inner black circles) that consists of a full-length negative strand and an incomplete (dashed lines) positive strand. The genome contains four promoters, two enhancer regions (Enh1, Enh2), and two direct repeats (DR1, DR2). The four ORFs are depicted by the colored arrows. During virus replication, the ~dsDNA genome is repaired into covalently closed circular (ccc) DNA, which serves as the template for viral transcription. The four major transcripts, shown as thin black outer arrows, are described in the text. Note that the *X* ORF (red) is present in all four HBV mRNAs.

HBV is a small DNA virus that infects hepatocytes of humans and chimpanzees. The 3.2-kb HBV genome is highly compact, and contains four overlapping open reading frames (ORFs) that encode seven proteins (Figure 2). Viral enhancers and promoters are embedded within ORFs.

The HBV life cycle is complex and has been previously described [10,36,37]. Briefly, following virus attachment and entry into hepatocytes, HBV is uncoated and the viral DNA genome is transported to the nucleus where cellular enzymes repair the ~dsDNA HBV virion DNA (~dsDNA) to form cccDNA. The latter is the template for viral transcription, generating mRNAs 3.5-, 2.4-, 2.1-, and 0.7-kb in size. The 3.5-kb pre-genomic RNA (pgRNA) is packaged into viral core particles, where it is reverse transcribed by the viral polymerase/reverse-transcriptase into the first strand, negative-sense DNA. The (−) strand DNA serves as the template for the synthesis of (+) DNA that is only 20%–80% unit length. The viral core particles either acquire an envelope and are released from the cell or recycle to the nucleus to begin a new round of replication. HBV cannot infect cells in culture, presumably due to the absence of the viral receptor.

The recent discovery of the sodium taurocholate co-transporting polypeptide (NTCP) as a functional receptor for both HBV and the hepatitis delta virus (HDV) [38] will likely benefit future HBV research [38,39,40]. The ability to study HBx functions in the context of virus infection, instead of in plasmid-transfected cells, may facilitate the identification of biologically relevant HBx functions that can be targeted to disrupt virus replication.

Most of what is known about HBV replication was learned from the related duck hepatitis B virus (reviewed in [41]) or from plasmid transfected cells, and validated in the highly related woodchuck hepatitis B virus (WHV) that infects woodchucks and causes a spectrum of disease similar to that in humans infected with HBV (reviewed in [42]). The WHV regulatory WHx protein is analogous to the human HBx protein, and is required for virus replication in woodchucks [43,44]. HBV replication is often studied in cells transfected with plasmid DNAs encoding wildtype HBV that is greater-than-genomic length [45], and leads to the production of virus that is infectious in human liver chimeric mice [46]. The contribution of HBx to virus replication is measured by comparing levels of virus produced by cells transfected with wildtype HBV (payw1.2, referred to as pHBV) [45] to HBV levels produced by cells transfected with an identical plasmid containing a point mutation preventing HBx expression (payw1.2*7, referred to as pHBVΔX) [47]. The HBx-deficient virus replication produced by pHBVΔX is restored to wildtype levels by cotransfection with a second plasmid encoding HBx [48]. The plasmid replication model is not an infection model, thus, it does not permit the study of virus-host interactions in the earliest steps of the virus life cycle.

### 3.2. The Regulatory HBx Protein

**Figure 3:**
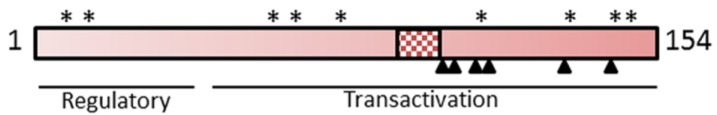
Schematic representation of HBx. The 154-aa HBx protein is conserved in all mammalian hepadnaviruses. Functional domains shown include the regulatory domain (aa 1–50), the transactivation domain (aa 50–154), and the DDB1 binding domain (aa 88–100; hatched area). The proteasome binding domain (aa 90–154) spans the DDB1 binding domain [69]. Conserved cysteine residues are shown by the asterisks (aa 7, 17, 61, 69, 78, 115, 137, 143, and 148) [70] and have an unknown function. Six conserved lysine residues, which may be targets for ubiquitination, are indicated as filled triangles (aa 91, 95, 113, 118, 130, 140) [70,71].

The smallest open reading frame of HBV encodes the 154 amino acid (aa) HBx protein (Figure 3). HBx is conserved among all mammalian hepadnaviruses but is absent from the avian hepadnaviruses [41]. HBx is produced at low levels during acute and chronic HBV infection, and localizes to both the nucleus and the cytoplasm [49,50,51]. Several comprehensive reviews summarize the many functions of HBx identified in plasmid transfection experiments [52,53,54,55,56,57,58,59]. There is general agreement that HBx exerts a two- to four-fold enhancement in viral and specific cellular gene expression assays that employ plasmid transfection [10,53]. HBx can bind with specific transcription factors, leading to the activation of cellular signal transduction pathways (reviewed in [57,60]). Since transcription factors that regulate HBV promoters and enhancers are present at varying levels in different cell lines, the activity of HBV enhancers and their stimulation of HBV promoters can vary significantly, depending on the cell line used. Comparison of HBV regulatory elements using the same constructs in transfected mouse cells* versus* in mouse liver* in vivo* (delivered by hydrodynamic injection) revealed that HBV enhancer effects on the *X* promoter were >100-fold higher* in vivo* than* in vitro* [61].

Several post-translational modifications of HBx have been reported, including acetylation, phosphorylation, and disulfide bond formation (reviewed in [62]), but the biologic importance of these modifications to HBx function remains unclear. HBx contains nine conserved cysteine residues (Figure 3, see asterisks), more than twice the number predicted for a mammalian protein of its size [63]. Two studies have concluded that the cysteines in HBx form four disulfide bonds, but the specific cysteines pairing in these bonds differ between studies [64,65]. These discrepant results may be due to the unfolding and refolding of HBx in the earlier study [64,65], which may have introduced spurious disulfide bonds. Site-directed mutagenesis to remove specific cysteine residues results in the loss of HBx function(s) (discussed in [65]). It is generally accepted that in eukaryotic cells, the formation of disulfide bonds occurs in the lumen of the rough endoplasmic reticulum and not in the reducing environment of the cytosol [66,67] where HBx is thought to localize. However, recent studies suggest that disulfide bonds may form in intermembrane space of the mitochondria and other compartments of the cytosol [68].

### 3.3. HBV Replication Requires HBx

The role of HBx in HBV replication has been studied in several model systems, and the requirement for HBx depends on the assay used. There is an absolute requirement for HBx in HBV infection models. Infection of human liver chimeric mice or HepaRG cells with virus particles encoding wildtype HBV that encode HBx leads to the production of new virus, while infection with virus particles containing HBV DNA with a point mutation that prevents HBx expression does not [46,72]. The absence of HBx in these infection models does not prevent the formation of cccDNA, indicating that HBx is not required for that early step in virus replication. However, viral RNA is not detected, supporting the idea that an important role for HBx in HBV replication is to enhance HBV mRNA formation from the cccDNA template. Indeed, HBx activates the expression of the four HBV transcriptional promoters by several mechanisms, including direct interaction with nuclear transcription components and activation of cytosolic signal transduction pathways (reviewed in [53]).

The relative contribution of HBx to the virus replication in HBV plasmid replication assays, in which virus replication is driven from relatively large inoculum of plasmid DNA, is less clear. Transfection of cells with a plasmid DNA encoding greater-than-genomic length wildtype pHBV [45] leads to the production of infectious virus [46]. Transfection of cells with the HBx-deficient pHBVΔX plasmid still produces HBV, albeit at significantly lower amounts [48]. Importantly the reduced HBV replication is restored to wildtype levels when a second plasmid encoding HBx is provided. The amount of HBx-independent virus replication varies depending on the assay used for measurement (discussed in [73]). These studies were extended* in vivo*. Intrahepatic inoculation of WHV genomes capable of expressing WHx produces viremia in woodchucks, whereas inoculation with WHV genomes containing mutations that prevent WHx expression either do not replicate [43,44] or are severely attenuated in their replication [74]. There was sufficient WHV expression from the DNA inoculum to induce protective immunity* in vivo*, even in the absence of HBx [74]. In summary, the contribution of HBx to virus replication is influenced by the assay used, with authentic HBV infection models providing the strongest evidence that HBx is needed for virus replication. Not surprisingly, the mechanism by which HBx functions in the HBV life cycle is hotly debated, due to experimental results and conclusions that are often influenced by experimental conditions. Important variables include the cell lines used, and the expression levels of HBx [73,75,76]. The limitations of HBV replication assays were recently reviewed and should be considered when interpreting data from non-infection models of HBV replication [73].

## 4. HBx Interactions with CRL4

Many viruses encode proteins that target the UPS (reviewed in [5,6,7,8,9]). Here, we summarize evidence that the HBx protein interacts with several components of the UPS, including DDB1, the CSN, and distinct subunits of the 26S proteasome. Understanding the functional impact of HBx interactions with the UPS remains a significant challenge due to the limited model systems in which to study HBV replication (described above).

### 4.1. HBx Binds Cellular DDB1

Several early HBx studies sought to identify cellular proteins that interact with HBx as a way of understanding HBx function in virus replication. Using a genetic approach, Lee *et al.* demonstrated an interaction between HBx and DDB1 [77], and this was confirmed by additional laboratories using a variety of approaches (reviewed in [78]). The minimal region of HBx needed for binding to DDB1 is aa 88–100 (Figure 3, hatched box). An X-ray crystallographic approach demonstrated that HBx binds to a pocket created by two β-propeller (BP) domains of DDB1, BPC, and BPA, with the carboxyl end of HBx protruding from the complex [79]. This protruding portion of HBx binds many different transcription factors needed for virus replication (reviewed in [80]). HBx binds to the same region of DDB1 to which DCAFs bind. Immunoprecipitation of CUL4, followed by Western blot analysis for HBx and DDB1, showed that all three proteins were in the same complex when HBx was expressed either as a GFP-fusion protein [79] or a non-epitope tagged HBx [81]. These results suggest that not only does HBx interact with DDB1 itself, but is also within the CRL4 complex. Importantly, this result suggests that HBx may modulate cellular pathways at the posttranslational level, an idea that represents a paradigm shift from the role of HBx as a viral transactivator.

The finding that the HBx proteins of all mammalian hepadnaviruses retain the ability to bind DDB1 suggests that this interaction is functionally important [82]. Indeed, the HBx-DDB1 interaction is required for virus replication in woodchucks and in transfected HepG2 cells. The intrahepatic injection of plasmid DNA encoding wildtype woodchuck hepatitis virus (WHV) led to virus replication in woodchucks, while injection of WHV DNA encoding WHx mutants unable to bind DDB1 did not [83]. A similar result was obtained in the HBV plasmid replication model. HepG2 cells transfected with pHBVΔX (that cannot express HBx) showed decreased HBV replication that could be restored to wildtype levels by cotransfection of plasmids encoding wildtype HBx but not HBx mutants that were unable to bind DDB1 [81,84]. These results convincingly demonstrate the importance of HBx-DDB1 to HBV replication. Given the known role of CRL4, HBx might act either to recruit antiviral factors for ubiquitination and degradation, or to alter the CRL4 substrate specificity.

### 4.2. HBx Is a Viral DCAF

**Figure 4:**
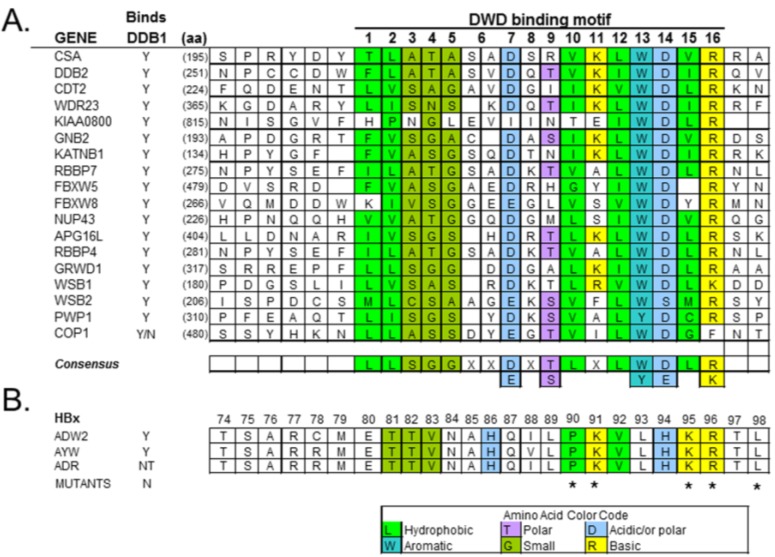
DDB1 binding motif found in DCAFs. (**A**), A DDB1-WD40 box (DWD) domain is present in some WD40-repeat-containing proteins [19] and mediates the binding of these DCAFs to DDB1. The 16-aa motif, estimated to occur in 79 genes in humans, is based on the biochemical/biophysical properties of the aa. (**B**), HBx sequences from three HBV subtypes show a similar DWD motif. Reproduced with permission from Reference [78]. Copyright 2008, Research Signpost.

Since DDB1 mediates its effects, in part, through interactions with DCAFs (discussed above, Section 2.1), the discovery that HBx shares many features with cellular DCAFs suggests a potential mechanism for altering CRL4 substrate specificity. Evidence for HBx being a viral DCAF comes from several independent laboratories. First, HBx contains the same 16-aa motif present in the ~79 cellular DCAFs that bind DDB1 (Figure 4) [78]. Second, X-ray crystallographic studies demonstrated that HBx interacts with DDB1 through this motif [79]. Third, immunoprecipitation of CUL4 followed by Western blot for HBx demonstrated that HBx is in the CRL4 complex [79,81]. Finally, the steady-state level of HBx is higher in ATX (HBx) transgenic mouse liver that expresses DDB1 than in ATX mouse liver with a flox-mediated DDB1 knockdown, indicating that HBx is not targeted for degradation by CRL4 but instead is stabilized [81]. These observations support the idea that HBx is a DCAF. However, the exact impact of HBx on DDB1 function is unknown. As a viral DCAF, HBx may influence CRL4 substrate specificity. Indeed, overexpressed HBx or WHx in transfected cells displaces cellular DCAF DDB2 [85] and DCAF9 [79] from DDB1. The effect of HBx on other DCAF-DDB1 interactions has not been reported. Indeed, the DDB1-DCAF profile of normal human hepatocytes is unknown. However, HBV infection and expression of the HBx viral DCAF is anticipated to alter the normal DCAF profile and to affect downstream substrates that are recruited for ubiquitination and degradation. Based on what has been reported for other viruses that target CRL4-DDB1 (Table 1), it is possible that more than one cellular pathway may be impacted by the HBx interaction with DDB1 (discussed in Section 6 below).

A second model for HBx-DDB1 in virus replication centers on the cccDNA, the known template for HBV transcription [10]. HBx does not bind DNA directly, and so the model predicts that DDB1 binds to the cccDNA and tethers HBx to modulate viral transcription [79,81]. In support of this idea, HBx protein can activate an HBV Enhancer luciferase reporter in transfected HepG2 cells, but HBx point mutant proteins unable to bind DDB1 lose this ability [79,81]. Thus, the HBx-DDB1 interaction is critical for viral transcription, and at least one function is a role in viral transcription.

### 4.3. HBx Binds the CSN Signalosome

Novel HBx binding partners were identified in plasmid transfected HEK 293T cells using tandem-affinity purification, coupled with mass spectrometry. In addition to known HBx targets DDB1 [77] and heat-shock protein 70 [86], this study also identified subunits 3, 4, and 5 of the CSN as HBx binding partners [87]. The CSN plays a regulatory role in CRL4 activity by removing NEDD8 from active CRLs, rendering the complex inactive (reviewed in [12]). The Epstein-Barr virus encodes the BPLF1 protein that has deneddylase activity and removes NEDD8 to facilitate virus replication by creating an S-phase-like environment in the cell [88] (Table 1). The possible effect of HBx on CSN function has not been reported. The HBx-CSN interaction might lead to a decreased NEDD8 on CUL4, to create an S-phase-like environment needed by HBV. Alternatively, the interaction of HBx with CSN might enhance the NEDD8 attachment to CUL4, thus, locking CRL4 in an active state that benefits virus replication.

## 5. HBx Interactions with the Proteasome

A genetic screen similar to that used to discover the HBx-DDB1 interaction also led to the discovery that HBx binds to PSMA7 (also known as XAPC7), an α-subunit of the 20S proteasome [89]. The same approach also identified PSMC1 (an ATPase-like subunit of the 19S regulatory component of the 26S proteasome) as a protein that interacts with HBx [69]. These results were confirmed and extended to include the demonstration of an interaction of HBx with another α-subunit (PSMA1) [90]. The carboxyl portion of HBx is important for this interaction [69].

The functional importance of the HBx-proteasome interaction was studied in HBV plasmid-based replication assays, and has yielded discrepant results. HepG2 cells were infected with recombinant adenovirus encoding wild-type HBV or an HBV containing a point mutation that prevented HBx expression (HBVΔX) and treated with proteasome inhibitors epoxomicin or MG132. The inhibitors had no impact on wildtype HBV virus levels, but restored the HBx-deficient virus replication to wildtype levels [91]. Similar results were obtained in HBV transgenic mice harboring either wildtype HBV genomes or HBV genomes with point mutations that abolished HBx expression [91]. However, another study of HBV transgenic mice treated with a different proteasome inhibitor (bortezomib) found a significant reduction in wildtype HBV replication [92]. The reason(s) for these different results are unclear, but may be due to the earlier time points used in the Bandi *et al.* study [92]. It is also possible that virus replication driven from the integrated transgene differs from that of the adenovirus-delivered HBV DNA. A clearer picture of the effect of proteasome inhibitors on HBV replication may best be addressed using an authentic HBV infection model, such as the human liver chimeric mouse [46] or HepaRG cells [72].

The original study to define the impact of HBx on the ubiquitination/degradation of cellular targets focused on two cellular proteins known to be degraded through ubiquitin-dependent proteasome pathway. HepG2 cells transfected with plasmid encoding HBx revealed an increased half-life of two known proteasome targets, c-*Jun* and Arg-β-galactosidase, suggesting that overexpressed HBx can block proteasome function [69]. It is not known if HBx has this inhibitory function in the context of HBV replication in hepatocytes, and when HBx is expressed at physiological levels. Given the multifunctional nature of HBx, it is likely that more than one cellular protein will be targeted as a result of the interaction of HBx with the proteasome. Several CRL4 DCAFs and their substrates have been identified that have roles in pathogenesis [93]. An HBx-derived peptide (aa 116–138) prevented binding of the proteasome activator protein PA28 to the PSMC7 subunit, and this is predicted to negatively impact the ability of the proteasome to process antigenic peptides for the purpose of MHC class 1 presentation [94].

## 6. Lessons from Other Viruses

Several viruses encode regulatory proteins that also target DDB1 and the CRL4 E3 ligase complex (Table 1). Information from these viral systems may provide insight into pathways impacted by the HBx interaction with DDB1 and are briefly summarized.

### 6.1. E3 Ligases and Innate Immunity

All viruses have evolved ways to overcome the innate immune response as part of their strategy to establish infection. The regulatory V protein of SV5 binds to DDB1 [95], leading to the recruitment and degradation of interferon-activated transcription factor STAT1 (signal transducer and activator of transcription 1) [96,97]. In a related strategy, the V protein of HPIV2 targets STAT2 for degradation while the V protein of mumps virus targets both STAT1 and STAT3 (reviewed in [98]). Additional DDB1-related strategies exist to overcome innate immunity. The Vpx protein of HIV2 promotes the degradation of cellular antiviral factor SAMHD1 by recruiting it to the DCAF1-DDB1 complex [99].

The ability of HBV to avoid activating the interferon response is well known (reviewed in [100]), although the mechanism by which this occurs is unclear. While there is evidence that HBx can inhibit the interferon response in cell culture [101,102,103], it remains unknown whether this occurs through a DDB1-related mechanism. It is important to address this question in a biologically relevant setting. Studies in cancer cell lines, such as HepG2, will provide an answer that may not represent what occurs in human hepatocytes during HBV replication.

**Table 1:** Viral proteins that bind CUL4-DDB1.

Virus family	Virus (protein) ^1^	Cellular pathway altered	Reference
*Paramyxoviridae*	Simian Virus 5 (V)	Innate immunity	[97]
	HPIV2 (V)	Innate immunity	[96]
	Mumps (V)	Innate immunity	[104]
*Hepadnaviridae*	HBV (HBx)	Unknown	-
*Retroviridae*	HIV-1 (Vpr)	Cell cycle	[105,106]
	HIV-2 (Vpx)	Cell cycle	[107]
*Flaviviridae*	HCV (NS3/4A)	Unknown	-
*Herpesviridae*	MHV-68 (M2)	Apoptosis	[108]
	EBV (BPLF1)	Cell cycle	[88]
	BHV-1 (VP8)	Unknown	-

^1^ HPIV2, Human Parainfluenza Virus 2; HIV-1, Human Immunodeficiency Virus type I; MHV 68, Murine Gammaherpesvirus; EBV, Epstein Barr Virus; HIV-2, Human Immunodeficiency Virus Type II; HCV, hepatitis C virus; BHV-1, Bovine Herpesvirus 1.

### 6.2. E3 Ligases and the Cell Cycle

Viral regulatory proteins often alter the cell cycle to favor virus replication. The HIV1 accessory Vpr protein binds CUL4-DDB1 [106] and mediates G2 cell cycle arrest [105,106]. Of interest, HBV infects resting (G0) hepatocytes but must induce G0/G1 cell cycle progression to activate the HBV reverse transcriptase function needed early in virus replication [109]. The ability of HBx to alter cell cycle progression is well known (reviewed in [110]), and it is possible that the HBx-DDB1 interaction alters factors that regulate cell cycle progression in HBV-infected hepatocytes. This could be to induce a G0/G1 progression or an S-phase-like state, or both.

### 6.3. E3 Ligases and the Damaged DNA Response

The activation of the damaged DNA response (DDR) is a common strategy used by viruses to provide factors needed for their replication. The M2 latency protein of murine gamma herpesvirus 68 binds to DDB1 and blocks DNA damage sensing activity in the cell, and down-regulates DNA damage repair [108]. Several studies have reported the ability of HBx to activate the DDR in immortalized mouse hepatocytes [111,112,113]. In plasmid-transfected HepG2 cells, HBx interferes with the NER pathway through a DDB1 mechanism [114,115]. The importance of the DDR to HBV replication is unknown. Activation of the DDR might be part of the innate immune response to the invading virus (reviewed in [116]). Alternatively, the DDR may be activated as part of the viral strategy to recruit cellular factors needed by the virus to repair the partially dsDNA genome. Since long-term DDR activation might be harmful to the cell, HBx may also act to inhibit the DDR. It will be important to study HBV and the DDR in infected human hepatocytes* in vivo* to establish the biologic relevance of the DDR to HBV.

## 7. Summary

The UPS plays a vital role in the overall health of the cell by marking proteins for degradation, and is targeted by many viruses as part of their strategy for virus replication. There is much evidence that the HBV regulatory HBx protein interacts with the UPS. However, our understanding of the biologic relevance of these interactions has been limited by the lack of convenient virus infection models. Only in recent years have significant advances been made in HBV model systems, and it is now possible to study HBx-UPS interactions in the context of virus infection. It is now appreciated that CRLs are deregulated in cancer, including HCC [31]. Defining how HBx modulates CRL4 may provide new approaches to interrupting chronic HBV infection and preventing the development of HCC.
